# Is the acute compartment syndrome diagnosed in snake bites true?: A review

**DOI:** 10.1097/MD.0000000000040008

**Published:** 2024-10-04

**Authors:** Carlos A. Cañas

**Affiliations:** a Universidad Icesi, CIRAT: Centro de Investigación en Reumatología, Autoinmunidad y Medicina Traslacional, Cali, Colombia; b Unit of Rheumatology, Fundación Valle del Lili, Cali, Colombia.

**Keywords:** antivenom, fasciotomy, intracompartmental pressure, snake envenoming

## Abstract

Envenomation caused by venomous snakes can induce clinical symptoms and signs resembling those of traumatic acute compartment syndrome (ACS), but it is uncertain whether its treatment guidelines are applicable or beneficial for ACS that is associated to snakebites. Nonetheless, recommendations for the diagnosis and treatment of trauma-induced ACS, particularly following fractures of the tibial diaphysis, are extrapolated to the diagnosis and treatment of snakebites despite evidence that the ensuing injuries are frequently not true ACS. Most biologists agree that the venom of snakes, especially those of the Crotalinae family (vipers) evolved to immobilize, kill, and initiate the digestion of their prey. The human local effects of viper envenoming are the result of digestion like those described in biological processes as acute pancreatitis, including secondary inflammatory and induction of reparative effects. The first-line treatment should focus on mitigation of venom-induced tissue digestion rather than surgery solution for “ACS-like” symptoms and signs. This type of analysis leads to questioning that treatment of ACS associated with snakebite cannot be extrapolated from recommendations formulated for trauma-induced ACS. The cornerstone of snake envenoming treatment is antivenom, and some clinical and experimental experiences start to show that surgical procedures frequently employed for trauma-induced ACS, such as debridement and fasciotomy, may be exaggerated and even deleterious in most viper bite envenoming.

## 1. Introduction

Snakebite envenoming is a medical condition associated with high morbidity.^[1]^ Epidemiological data are fragmented and inaccurate, especially in developing countries where many victims do not go to health centers or hospitals and instead rely on traditional treatments. Nonetheless, best available data suggest that between 4.5 and 5.4 million people are bitten by snakes each year and that roughly 1.8 to 2.7 million bite recipients develop clinical diseases, while between 81,000 and 138,000 die of complications.^[2]^ Although mortality risk after envenomation is low, secondary complications are observed in 10% to 44% of cases.^[3]^

Snakebite injuries are caused by the actions of various components of the venom that have evolved to immobilize, kill and in some cases initiate the digestion of prey.^[4]^ The digestive processes are mediated primarily by proteases and lipases that ultimately induce varying degrees of membrane rupture, cytolysis, and extracellular matrix degradation. Accidental envenoming caused by defensive viper bites typically manifest with edema, bleeding, and varying degrees of necrosis in the skin, subcutaneous tissue, and deep structures such as fascia, muscles, and nerves.^[5]^ Additionally, systemic effects can worsen local tissue conditions, including hypotension caused by bradykinin-enhancing peptides and changes in coagulation caused by fibrinogen-degrading enzymes, plasminogen activators, prothrombin activators, factor V activators, prothrombinase complex formation factor inhibitors, thrombin activator inhibitors, phospholipases, and protein C.^[6]^

The cornerstone of treatment for venomous snakebites is antivenom, which consists of hyperimmune immunoglobulins obtained from venom-injected animals,^[7]^ a formulation that has remained largely unchanged since the first description by Albert Calmette in 1896.^[8]^ If antivenom is applied in a timely manner, complications are usually minimized,^[9]^ so complications following snakebites are largely attributable to a lack of antivenom availability, underdeveloped healthcare infrastructure, and the low socioeconomic status of the patient.^[10]^

One of the most feared complications of snakebite is attributed to acute compartment syndrome (ACS). However, it remains controversial if typical symptoms of snakebite are indicative of an ACS similar to that induced by other types of traumatic tissue injury and thus if guidelines established for traumatic ACS are relevant to the treatment of snakebite. Regardless of etiology, ACS is characterized by increased pressure from edema and bleeding in anatomical spaces confined by a lack of fascial distensibility. This enhanced pressure in turn reduces blood perfusion, resulting in hypoxia and necrosis of muscle, nerve, or skin, pathogenic processes that are progressively aggravated with the passage of time.^[11]^

The diagnosis, treatment, and outcomes of ACS associated with fractures are well documented, and both monitoring and management techniques have advanced considerably since the first description more than 100 years ago.^[12,13]^ Alternatively, the diagnostic features, monitoring targets, and optimal treatment options for ACS related to snakebites are still debated. A “false ACS” condition may be diagnosed based on shared clinical manifestations.

The objective of the current manuscript is to highlight the controversies surrounding ACS caused by snake envenoming, the contributions of digestive processes to snakebite pathobiology, and the implications of tissue digestion to treatment choice. In contrast to trauma-induced ACS, snakebite shares pathomechanisms with acute pancreatitis, a condition in which degradative enzymes are released that damage intraabdominal tissues. However, due to the transient nature of this damage, we generally recommend conservative treatment with antivenom and using best clinical judgment before considering fasciotomy, debridement, or amputation.

### 1.1. ACS associated with trauma

The most common as well as the best described and studied form of ACS is that developing in the legs after tibial diaphysis fracture, and the most feared consequences of this condition are muscle and peripheral nerve ischemia. The diagnosis of trauma-induced ACS is based mainly on clinical presentation. The most consistent symptom is pain, which increases disproportionately relative to the underlying injury, is resistant to standard analgesics, and is aggravated by passive stretching of the muscles in the affected compartment. Patients may also present with paresthesia or hypesthesia. Pathological compartments may be tense on palpation, pallid, and lack a pulse,^[14]^ consistent with local circulation block due to high intracompartmental pressure and ensuing ischemia.

Muscle necrosis has been detected within 3 hours and neural necrosis roughly 8 hours after the precipitating event. Thus, traumatic ACS must be treated within about 6 hours to prevent sequela of peripheral ischemic nerve damage such as loss of sensation, motor deficits, and chronic pain, and of muscular damage such as contractures, chronic stiffness, and deformities.^[15]^ Special care should be taken to recognize ACS in patients with regional block, intubation, sedation, or altered state of consciousness.^[16]^ In addition to bone fracture, other causes of ACS nonfracture-related ACS) include soft tissue trauma, reperfusion after ischemia, prolonged use of tourniquets or tight splints, and bleeding disorders.^[17]^

### 1.2. ACS attributed to snakebites

The local injuries caused by many venomous snakebites, particularly bites by members of the Crotalinae family such as copperheads, moccasins, and rattlesnakes, are the result of enzymatic damage to tissues (skin, subcutaneous fat, fascia, muscles, nerves, and blood vessels). These injuries may manifest clinically as edema, pain, paresthesias, hypoesthesias, blisters, ecchymosis, bleeding, cyanosis, and black discoloration associated with necrosis. Alternatively, some forms of snake envenomation, such as from the genus *Crotalus*, can manifest with little pain due to the concomitant activity of neurotoxins.^[18]^ Depending on the severity of envenoming, the edema may persist locally or spread. The clinical manifestations of snakebite exhibit highly variable onset, ranging from 6 to 72 hours.^[19]^ While these symptoms are similar to those of traumatic ACS, the specific clinical course can differ substantially.^[20]^ For instance, the muscle damage associated with snakebites starts almost immediately, progresses due to enzymatic activity and myotoxicity, and is eventually exacerbated by ischemia due to blood vessel damage. These combined pathogenic processes may lead to true ACS or a cluster of symptoms resembling ACS. The distinction is critical because fasciotomy, a therapeutic approach used frequently for trauma-induced ACS, may actually increase the morbidity of snakebites.^[21]^ In addition to these early events, snakebites may induce delayed secondary infections (usually within about 48 hours) from resident bacteria in the animal’s mouth.^[22]^ Deep vein thrombosis^[23]^ or necrotizing fasciitis^[24]^ are rarely associated with thrombosis.

Estimating the frequency of (true) ACS from snakebites is difficult and many patients are misdiagnosed. The majority of Asian, European, and Latin American studies have report ACS in 4% to 15% of snakebite cases,^[25–27]^ but others have reported this complication in only around 1% of cases, including a series reporting 21 ACS cases among 1604 snake envenoming incidents over 9 years based on records from the Toxicology Investigators Consortium NASBR of the United States between January 1, 2013 and December 31, 2021.^[28]^

### 1.3. Diagnosis based on measure of intracompartmental pressure

A pulse pressure drop (ΔP, or diastolic blood pressure) below 30 mm Hg is considered inadequate for local perfusion in an extremity and indicative of ACS, while an intracompartmental pressure >30 mm Hg is considered a risk factor for ACS.^[29]^ Local ΔP monitoring is recommended for the diagnosis of trauma-induced ACS. However, the time required for low perfusion to cause irreversible ischemic damage is unclear and determining the precise time of ACS onset is dependent on numerous factors, so “ischemia time” is usually only an educated guess in clinical settings.^[30]^ Sheridan and Matsen found that 68% of patients treated within 12 hours recovered normal lower extremity function compared with only 8% treated after 12 hours,^[31]^ suggesting that 12 hours is the maximum tolerable ischemic period. Janzing and Broos found that symptoms alone identified ACS correctly with 89% specificity and 67% sensitivity, while a ΔP below 30 mm Hg identified ACS with 65% specificity and 89% sensitivity.^[32]^ Therefore, compartment pressure monitoring should be used to confirm clinical suspicion of ACS. Moreover, this may be achieved noninvasively by infrared monitoring of tissue perfusion.^[33]^

The utility and necessity of intracompartmental pressure measurement for snakebite is controversial. Invasive methods such as the installation of needles increase the risk of further tissue damage and infection. Moreover, there are no established treatment standards based on ΔP. While one algorithm based on clinical findings complemented by intracompartmental pressure measurement included a recommended threshold of 45 mm Hg for fasciotomy,^[34]^ a case study reported that a 17-month-old child with compartment pressure reaching 85 mm Hg was successfully treated with antivenom and supportive care without fasciotomy.^[35]^ This case underscores the primacy of antivenom as first-line treatment and suggests that measuring intracompartmental pressure is of limited value for ACS associated with snakebite. Alternatively, noninvasive methods for the evaluation of possible ACS such as ultrasound and assessment of diastolic retrograde arterial flow^[36]^ may improve diagnostic accuracy without increasing the risk of treatment-related adverse events.

### 1.4. Treatment

The primary nonconservative treatment for trauma-induced ACS is fasciotomy, which must be performed before the onset of irreversible tissue necrosis. Although, this decision can be difficult based on clinical parameters,^[37]^ delayed fasciotomy increases the risk of irreversible muscle and nerve damage.^[38]^ Alternatively, antivenom is the first-line treatment for venomous snakebites, with the dose administered according to the degree of envenoming.^[39]^ If antivenom is used in a timely manner, possible ACS can be prevented, and fasciotomy avoided.^[40]^ Patients with snake envenomation should remove tight clothing and jewelry from injured extremities to prevent tightening due to swelling. Additionally, the application of tourniquets, ice packs, irritating substances, and incisions at the bite site should be avoided.

Again, there are no accepted standard indications for fasciotomy following snakebites as such invasive interventions are frequently accompanied by short-term complications like bleeding, infection, sepsis, kidney failure, and even death as well as long-term complications such as deformities, neuromuscular sequelae, and open wounds that are slow to heal.^[41,42]^ Fasciotomies should be limited to strict cases where a true ACS is present, based on clinical judgments.^[21,43–45]^ There are reported algorithms based on large series of patients, that allow adequate selection of which patients should undergo this procedure.^[34]^

### 1.5. Parallels between snake envenomation and acute pancreatitis

The pathobiology of snakebites by venomous species such as of the Crotalinae family is best understood as a digestive process leading to cytotoxicity, extracellular matrix destruction, secondary inflammation, and eventually tissue repair.^[46]^ Cytotoxicity is also probably made worse by bleeding and ischemia, where a true component of ACS would be involved in its pathogenesis. The pathological effects of venom, depending on the type of snake species, can be systemic (neurotoxicity, coagulopathy, and hemolysis) or local (e.g., edema, cytolysis, and destruction of extracellular matrix due to digestive enzymes in venom). Depending on the type of species, these biological effects may vary. For example, viper envenomations including Crotalinae, are more hemotoxic,^[47,48]^ and *Daboia russelii* and *Bitis arietans* more cytotoxicity.^[49,50]^ Common venom enzymes include phospholipase A2 (PLA2), which generates inflammatory factors involved in edema, tissue damage, and myotoxicity. Metalloproteases can induce extracellular matrix destruction and dermonecrosis, l-amino acid oxidases are implicated in tissue damage, serine proteases and type C lectins are able to induce tissue damage and hemorrhagic diathesis, serine proteases can exacerbate tissue damage, and disintegrins are known to induce the detachment of cells from extracellular matrix.^[51]^ Subsequently, an inflammatory response is triggered involving the recruitment of neutrophils, macrophages, and lymphocytes and further inflammatory cytokine cascades that sustain the inflammatory process.^[52]^ Under most conditions, these pathogenic processes are followed by anatomic and functional recovery, especially in cases with prompt antivenom treatment.^[53]^

Conversely, acute experimental pancreatitis is associated with local cytolysis, extracellular matrix degeneration, and hemorrhage.^[54]^ An important trigger for this condition is intracellular calcium dysregulation, resulting in conversion of trypsinogen to the active protease trypsin, which damages the vascular endothelium and enhances tissue infiltration of neutrophils, macrophages, and lymphocytes.^[55]^ Trypsin also activates MMP-9, potentially leading to a positive feedback loop that drives further tissue digestion.^[56]^ In addition, damaged pancreatic acinar cells release pancreatic group I PLA2, while ensuing enzymatic activities lead to activation of nonpancreatic group II PLA2 (PLA2-II), both of which drive further tissue injury and inflammation. In particular, PLA2-II activity is central to the systemic inflammatory activity associated with acute pancreatitis, the membrane-associated C-type lectin-1 (Dectin-1) pathways are activated in macrophages,^[57]^ the protease A disintegrin and metalloproteinase 17 trigger additional inflammatory responses through shedding of bioactive inflammatory mediators,^[58]^ and serine proteases including trypsin are further activated. The nascent tissue damage caused by these enzymatic processes in turn triggers the release of multiple pro-inflammatory cytokines.^[59]^ Once these pathogenic processes subside, reparative processes begin involving transforming growth factor-beta and insulin-like growth factor-1, and pancreatic stellate cells contribute to fibrogenesis and tissue remodeling.^[59]^

## 2. Conclusions

Snake envenoming triggers pathogenic proteolytic and inflammatory cascades resembling those detected in acute pancreatitis but distinct from those of traumatic ACS. Thus, the relevant treatment indications and targets for venomous snakebites differ from those recommended for traumatic ACS. While debridement and fasciotomy may be necessary for traumatic ACS of the limb, these can be highly detrimental to the outcome of envenomation. The treatment of snakebites should be conservative, and its cornerstone is the antivenom. The reparative processes induced after termination of the pathological processes triggered by venom (which is accelerated by antivenom) begin with the recovery of tissue structure and function, including the restoration of muscle and nerve activity. Therefore, surgical treatments such as debridement and fasciotomy that may damage these tissues should be reserved for severe cases and those for which treatment is delayed by several hours. Neither the diagnosis nor the management of ACS associated with snakebites can be extrapolated from the recommendations for trauma-induced ACS. Additional preclinical and clinical studies are warranted to develop relevant management recommendations for snakebite with and without ACS.

## Acknowledgments

Tecnoquímicas for its support of CIRAT.

## Author contributions

**Conceptualization:** Carlos A. Cañas.

**Formal analysis:** Carlos A. Cañas.

**Methodology:** Carlos A. Cañas.

**Writing – original draft:** Carlos A. Cañas.

**Writing – review & editing:** Carlos A. Cañas.
